# In Vitro Formation of β Cell Pseudoislets Using Islet-Derived Endothelial Cells

**DOI:** 10.1371/journal.pone.0072260

**Published:** 2013-08-28

**Authors:** Michael G. Spelios, Lauren A. Kenna, Bonnie Wall, Eitan M. Akirav

**Affiliations:** 1 Research Institute, Islet Biology, Winthrop-University Hospital, Mineola, New York, United States of America; 2 Stony Brook University School of Medicine, Stony Brook, New York, United States of America; Broad Institute of Harvard and MIT, United States of America

## Abstract

β cell pseudoislets (PIs) are used for the in vitro study of β-cells in a three-dimensional (3-D) configuration. Current methods of PI induction require unique culture conditions and extensive mechanical manipulations. Here we report a novel co-culture system consisting of high passage β-cells and islet-derived endothelial cells (iECs) that results in a rapid and spontaneous formation of free-floating PIs. PI structures were formed as early as 72 h following co-culture setup and were preserved for more than 14 d. These PIs, composed solely of β-cells, were similar in size to that of native islets and showed an increased percentage of proinsulin-positive cells, increased insulin gene expression in response to glucose stimulation, and restored glucose-stimulated insulin secretion when compared to β-cells cultured as monolayers. Key extracellular matrix proteins that were absent in β-cells cultured alone were deposited by iECs on PIs and were found in and around the PIs. iEC-induced PIs are a readily available tool for examining β cell function in a native 3-D configuration and can be used for examining β-cell/iEC interactions in vitro.

## Introduction

The islets of Langerhans are three-dimensional (3-D) structures which contain insulin-producing β-cells. Disruption of the islet structure alters β cell function by inducing β cell dedifferentiation and impairing β cell survival [1], [2], [3]. The formation of 3-D β cell aggregates, or pseudoislets (PIs), is useful for the study of β cell biology. β-cells in PIs show improved function as measured by increased insulin production and improved glucose-stimulated insulin secretion (GSIS) [4], [5], [6], [7], [8], [9], [10]. These effects are mediated in part by the formation of a 3-D configuration which enhances β-cell – cell contact [5], [9], increases calcium signaling [11], and preserves extracellular matrix (ECM) proteins [12]. Despite their usefulness, PI generation requires extensive cell handling and may take several days to form (7–14 d). Current methods for induction of PIs include the use of mechanical manipulations such as stirred cell suspension cultures [8], culturing of β-cells on gelatin coated plates [4], and hanging drop cell cultures [13].

The islet endothelium plays a critical role in β cell function and survival [14]. Changes in islet endothelial cell (iEC) density and activation are associated with altered β cell function under physiological and pathological conditions. The control of β cell function and mass is partially mediated by the ability of iECs to produce pro-β cell factors [15] and support islet structure via the deposition of ECM proteins such as collagen IV (col-IV) and laminin [16], [17]. In isolated human islets, the addition of ECM proteins delays β cell dedifferentiation while maintaining insulin expression [18].

In this report we describe a straightforward and rapid method for inducing free-floating PIs by co-culturing iEC and β cell insulinoma lines. Newly formed PIs are positive for ECM proteins produced by iECs and show improved insulin production, insulin sensing, and GSIS when compared with monolayer cells. iEC-induced PIs are a readily available tool for examining β cell function in a native 3-D configuration and can be used for examining β-cell/iEC interactions in vitro.

## Materials and Methods

### Cell lines

MS1 murine iECs [19] were obtained from the American Type Culture Collection (Manassas, VA). βTC3 murine insulinoma cells were previously described [20] and were a kind gift from Dr. Kevan Herold (Yale University, New Haven, CT). High passage, (40–55) βTC3 cells were chosen to examine the effect of PI formation on βTC3 cells with poor insulin production and GSIS.

### Cell cultures and PI formation

βTC3 cells were cultured in Dulbecco's Modified Eagle's Medium (DMEM) containing 25 mM glucose and supplemented with 4.4 mM sodium bicarbonate, 15 mM HEPES, 1% penicillin/streptomycin/neomycin mixture, 15% heat-inactivated horse serum, 2.5% FetalClone II, and 1% Eagle's Minimum Essential Medium with nonessential amino acids. MS1 cells were also cultured under hyperglycemic conditions in DMEM modified with 5% heat-inactivated fetal bovine serum (FBS), 1% antibiotic mixture, and 0.25 µg/mL amphotericin B. All cell cultures were kept at 37°C in a 5% CO_2_ in air humidified atmosphere.

For PI formation, β-cell/iEC co-cultures were prepared by seeding βTC3 (2*10^5^ cells/well) and MS1 (6*10^5^ cells/well) in a 6-well tissue culture plate. The co-cultures were maintained for 7 d in DMEM supplemented with 25 mM glucose, 10% heat-inactivated FBS, 1% antibiotic mixture, 0.25 µg/mL amphotericin B, and 1 mM sodium pyruvate. β cell monolayers were propagated concomitantly under the same growth conditions as the βTC3/MS1 co-cultures.

### FACS analysis

Determination of insulin positivity and cell death was done by fluorescence-activated cell sorting (FACS) analysis using a C6 flow cytometer (BD Biosciences, Ann Arbor, MI). For insulin, βTC3 monolayer and dispersed PI cells were permeabilized (Fix & Perm, Life Technologies, Grand Island, NY) and stained with either biotin-conjugated monoclonal mouse anti-proinsulin antibody (Clone 253627, no reactivity with mature insulin, R&D Systems, Minneapolis, MN) or biotin-conjugated isotype control. Cy3-conjugated streptavidin (Jackson ImmunoResearch Laboratories, West Grove, PA) was used to detect insulin positive cells. To determine βTC3 viability, non-permeabilized cells were stained with 7-AAD (BD Biosciences, San Jose, CA).

### Immunofluorescence

Monolayer MS1 and βTC3 cells were grown on glass bottom Petri dishes coated with poly-D-lysine. Prior to staining, monolayer cells were washed in PBS and fixed in 2% PFA. Free floating PIs were washed and fixed in suspension. Following fixation, monolayer cells and PIs were stained with primary antibodies to insulin (Invitrogen, Carlsbad, CA), col-IV (Abcam, Cambridge, MA ), laminin (Abcam, Cambridge, MA ), BS-1 (Sigma Aldrich, Saint Louis, MI), or cleaved caspase-3 (Cell Signaling Technology, Danvers, MA) along with 4′,6-diamidine-2-phenylindole dihydrochloride (DAPI). Fluorescent labeled secondary antibodies were used for detection of primary antibodies (Jackson Immunoresearch, West Grove, PA). Some specimens were stained using secondary antibodies only and used as non-specific staining controls. Stained monolayer cells and PIs were analyzed using a Nikon Eclipse Ti confocal microscope (Nikon, Melville, NY). Z-stack confocal imaging and reconstruction were used to evaluate PI structure and characterize βTC3 cell configuration inside the islet core.

### Insulin release studies

β cell monolayers were trypsinized after 5 d and subcultured for 48 h in a 96-well tissue culture plate at a cell density of 3*10^4^/well. PIs received fresh medium on the fifth day of co-culturing, and co-cultures were maintained for an additional 2 d. The monolayers were subsequently deprived of glucose for 2 h followed by 1 h incubation in a balanced salt solution [4] with varying glucose amounts. Glucose deprivation of the PIs was carried out in a Petri dish after which the PIs were distributed in a 96-well tissue culture plate and exposed to the same range of glucose concentrations as the monolayers. Secreted insulin was quantified from the cell supernatants using ELISA kit (Crystal Chem Inc., Downers Grove, IL) according to the manufacturer's instructions.

### RNA isolation and quantitative RT-PCR

Total RNA was purified from cell lysates using an RNeasy Mini Kit (Qiagen, Valencia, CA). cDNA was synthesized with a Transcriptor High Fidelity cDNA Synthesis Kit (Roche, Indianapolis, IN) and a MyCycler thermal cycler (Bio-Rad, Hercules, CA) under the following conditions: RNA denaturation at 65°C for 10 min; cDNA synthesis at 45°C for 30 min, and reverse transcriptase inactivation at 85°C for 5 min. Primers used for semi-quantitative PCR were as follows: ***Col-IV:*** F-TGGGCGAGGGACATGCAATTACTA, R-ACATCGGCTAATACGCGTCCTCAA; ***Laminin α1:*** F-GACCGCCATGCCGATTTAGC, R-GACCGCCGTGTTGTTGATGC; ***Laminin α5:*** F-CCCTGGGGCCTTGAACTTCTCCTACTC, R-GCATTGCGCCGATCCACCTCAG; ***Laminin β1:*** F-ACCAGACGGGCCTTGCTTGTGAAT, R-AGTTGTGGCCCGTGGTGTAGTCCTG; ***β-Actin:*** F-GTGGGCCGCCCTAGGCACCA, R-CTCTTTGATGTCACGCACGATTTC.

Gene quantification was performed using SYBR Green I Master reaction mix (Roche, Indianapolis, IN) and a LightCycler 480 II system (Roche, Indianapolis, IN). Real-time PCR conditions were as follows: 95°C for 5, 45 cycles of 95°C for 1 min, primer annealing at 67°C (for insulin) or 60°C (all other genes) for 30 s, and target elongation at 72°C for 45 s. β-actin was used as a housekeeping gene in all reactions. Primers: ***Insulin 1*** *:* F-ACCTGGAGACCTTAATGGGCCAAA, R-ATGACCTGCTTGCTGATGGTCTCT; ***Bcl-2***: F-TTTGATTTCTCCTGGCTGTCTCTG, R-ATATTTGTTTGGGGCAGGTTTGTC; ***Bcl-xl***: F-GTTCTCTTGGCCTCAAAACTCACA, R-ACACATCAAAACCAAGGCAAGCTA; ***Ki67***: F-GCAAACCCTCACACTTGGAGAA, R-TGCCACGAGTAACATATATTGCTGG; ***β-Actin***: F-GCAAGTGCTTCTAGGCGGAC, R-AAGAAAGGGTGTAAAACGCAGC.

### Statistical Analysis

Data are expressed as mean ± SEM. The differences between means and the effects of treatments were analyzed by Student's t-test, one-way ANOVA with Tukey's post hoc test, or two-way ANOVA using Prism 5 (GraphPad software) where appropriate. Differences between treatments were considered significant at p<0.05.

## Results

### MS1 induce a spontaneous formation of free-floating PIs

**Figure 1:**
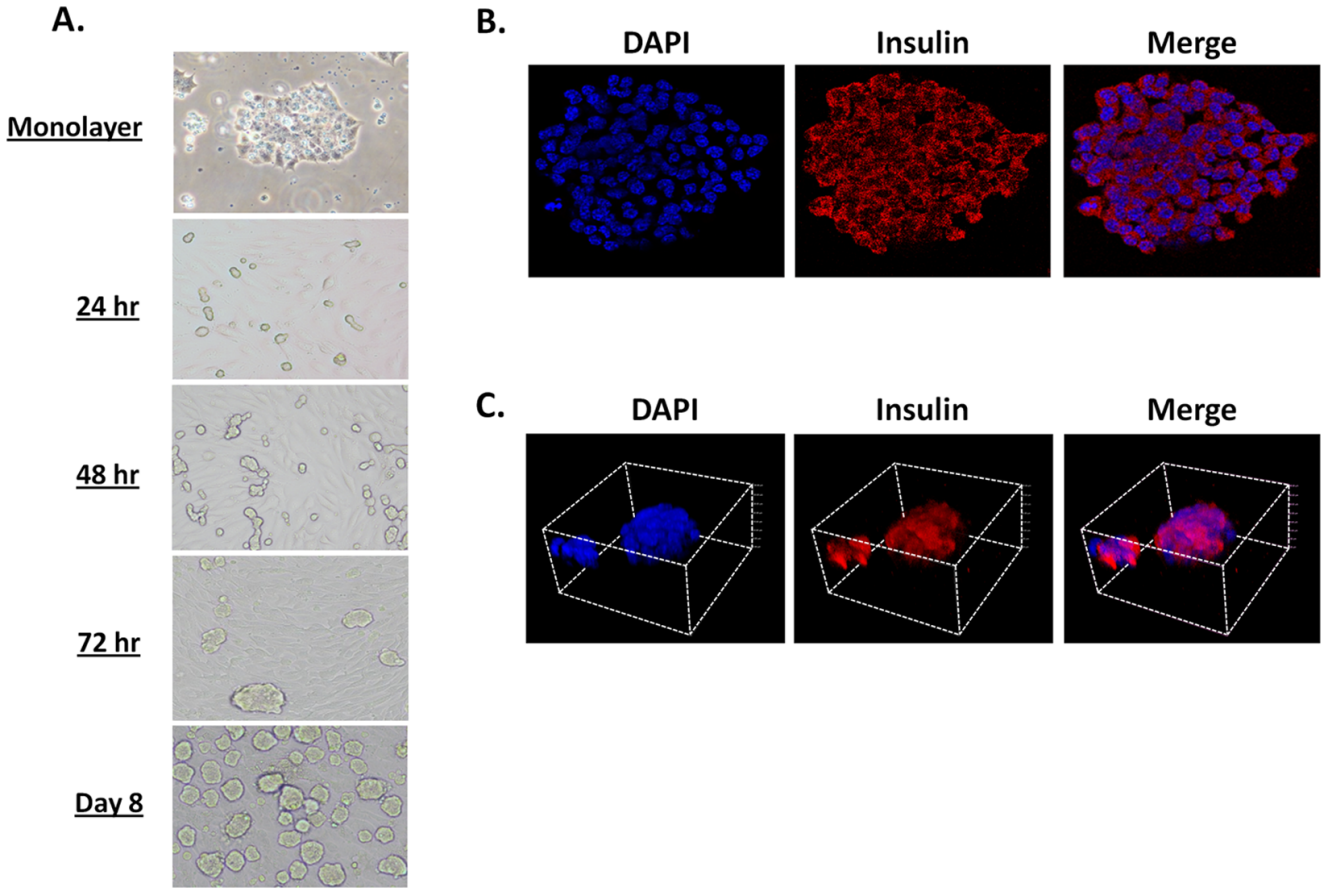
MS1 induces a spontaneous formation of free-floating insulin-positive PIs. ***A.*** Light and phase contrast microscopy showing the formation of βTC3 PIs over 8 d. ***B.*** IF staining of βTC3 monolayers. Blue- DAPI, Red- Insulin and merge. ***C.*** 3-D reconstruction of z-stack confocal images of a representative PI. Blue- DAPI, Red- Insulin and merge.

The islet endothelium plays an important role in the formation of the islet structure and β cell function [21]. To examine the effects of iECs on β-cells, we mixed MS1 iECs (6*10^5^) together with βTC3 insulinoma cells (2*10^5^). βTC3 cultured in the presence of MS1 cells formed cell clusters as soon as 24 h, which spontaneously detached by 72 h forming free-floating PIs (Figure 1A). PI density was increased by day 8 in culture (Figure 1A), showing a spheroid structure ranging from 50 to 300 µm in size, similar to native murine islets [22]. In contrast, βTC3 cells cultured alone remained adherent and failed to form PIs (Figure 1A). Monolayer βTC3 cells stained positive for insulin (Figure 1B). Similarly, PIs showed strong insulin staining, albeit in a 3-D structure (Figure 1C). Interestingly, MS1 iECs were not detected in or around the PIs as indicated by the absence of CD31 and BS1 staining (data not shown). Taken together, these results demonstrate a novel method for the rapid and spontaneous formation of free-floating PIs by co-culturing MS1and βTC3 cells in vitro.

### MS1-induced PIs show improved insulin production and glucose sensing when compared with monolayer cells

**Figure 2:**
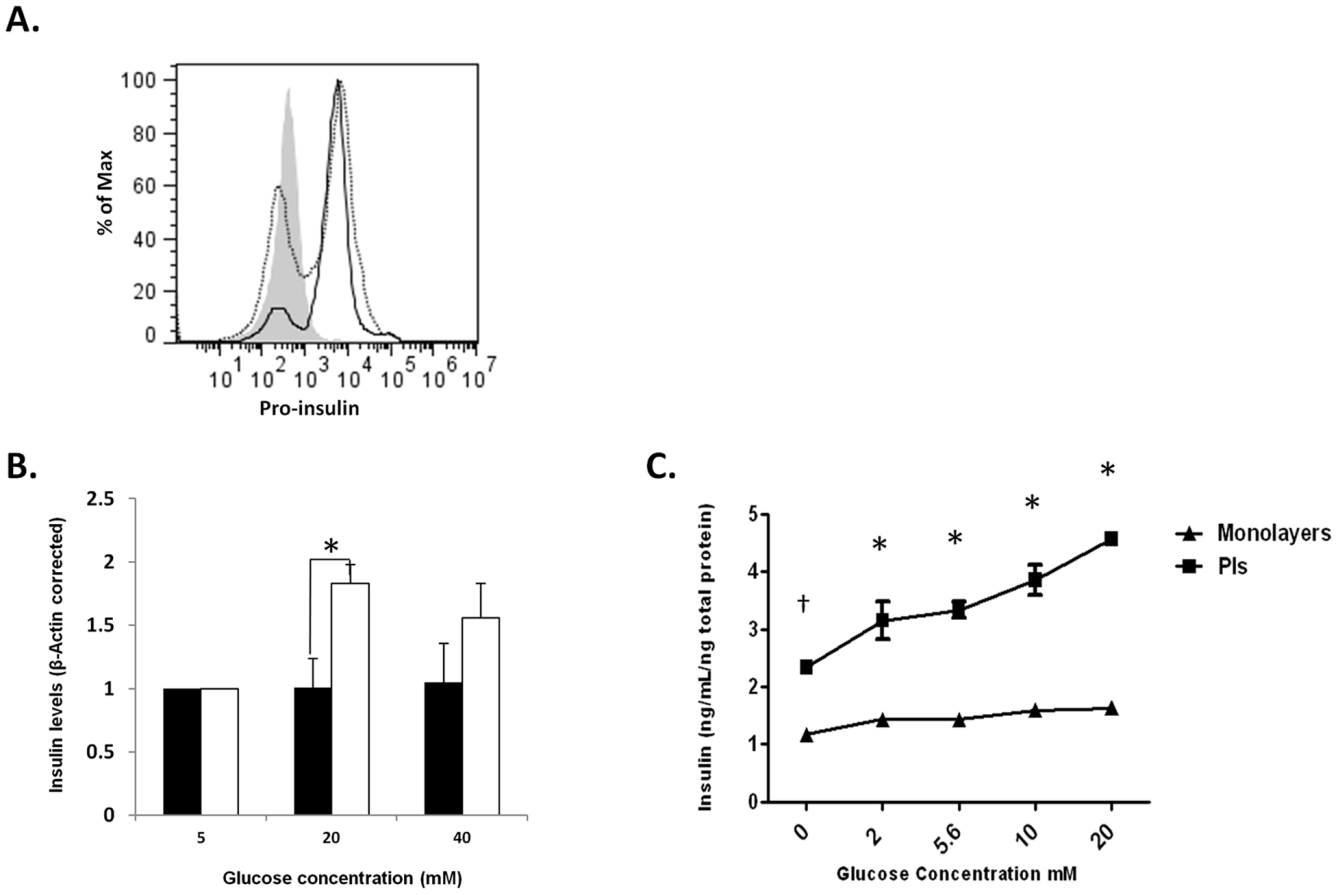
Baseline and glucose stimulated insulin expression and secretion are enhanced in MS1-induced PIs. ***A.*** FACS staining for proinsulin expression in monolayers (dotted line) and PIs (solid line). Grey histogram- isotype control. MFI: mono = 6579, PIs = 7486 ***B.*** Quantitative RT-PCR analysis of insulin 1 expression in βTC3 monolayers (closed bars) or PIs (opened bars). Data represents an average of 3 independent experiments. *p<0.025. Monolayers and PIs were cultured for 8 d, washed with media lacking glucose, and incubated with escalating glucose levels for 6 h. ***C.*** Insulin ELISA analysis of supernatant from βTC3 monolayers and PIs. Experiment represents three independent repeats. N = 3 per group. Two-way ANOVA analysis**.** ^†^p<0.001, *p<0.0001.

Previous reports show improved insulin production and GSIS following PI formation in high passage insulinoma cells [4]. We compared the ability of high passage monolayer and PI βTC3 to produce and release insulin in response to glucose stimulation. FACS analysis revealed an increase in the percentage of proinsulin-positive cells in MS1-induced PIs when compared with monolayer βTC3 cells (Figure 2A, proinsulin positive cells: monolayers = 52.7%, PIs = 76.4%). PIs incubated at high glucose concentrations (20 and 40 mM) showed higher insulin transcription as measured by increased levels of insulin mRNA when compared with βTC3 monolayers (Figure 2B, p<0.025). Moreover, analysis of insulin secretion in response to glucose stimulation showed increased baseline insulin and GSIS in PIs but not monolayer βTC3 cells (Figure 2C, ANOVA p<0.028). Collectively, these data demonstrate an improved insulin production and enhanced glucose responsiveness of βTC3 cells cultured in the presence of MS1 cells, consistent with previous reports [4], [5], [9].

### PIs are positive for laminin and col-IV and do not show increased apoptosis compared to monolayer cultures

**Figure 3:**
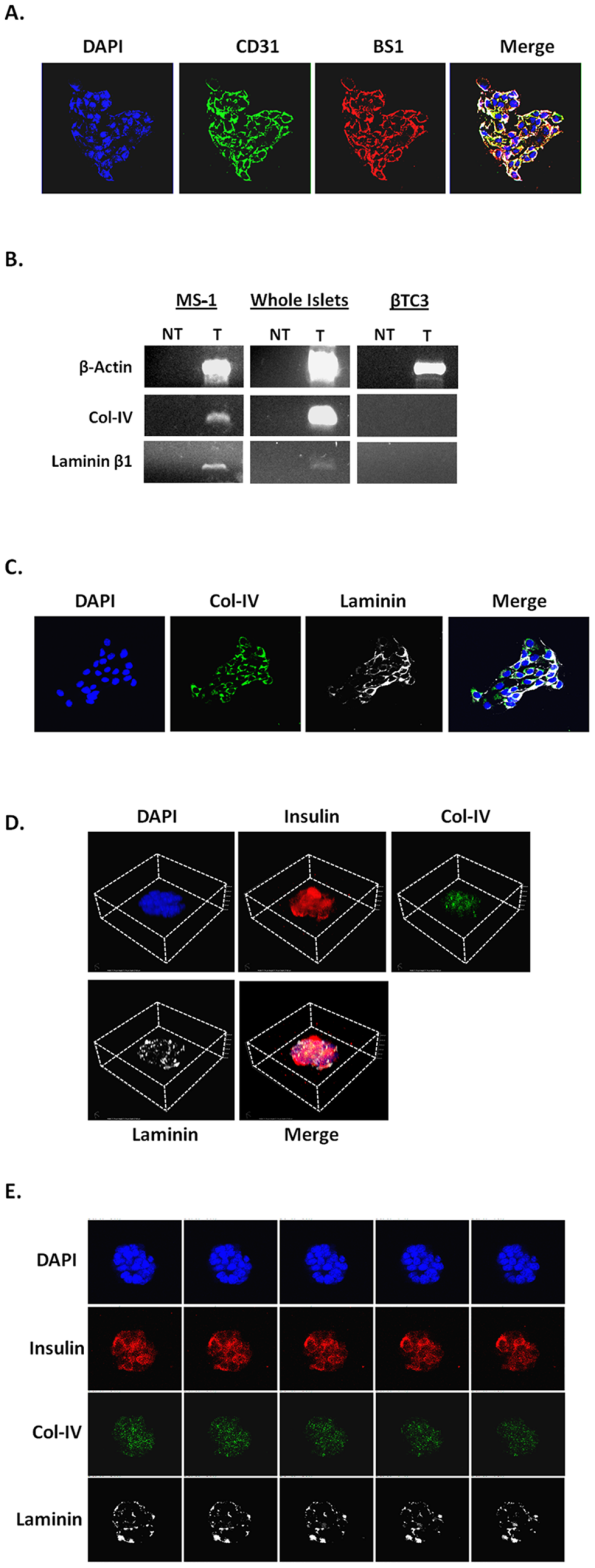
Col-IV and laminin are detected in and around the PI. ***A.*** IF staining of MS1 cells. Blue-DAPI, Green-CD31, Red-BS1 and merge. White/Yellow represents double positive cells. ***B.*** RT-PCR for laminin β1 and col-IV in MS1, whole murine islet preps, and βTC3 cells. Laminin α1 and α2 were not detected (data not shown). ***C.*** IF staining of MS1 cells. Blue- DAPI, Green- col-IV, White-Laminin, and merge. ***D.*** 3-D reconstruction of z-stack imaging of an 8 d old PI. ***E.*** Non-consecutive z-stack confocal images of a PI. Blue- DAPI, Red- Insulin, Green- col-IV, White- laminin.

ECM proteins, such as laminin and col-IV, are produced by iECs and are an integral part of the islet structure [16]. We tested whether MS1 cells express markers of native iECs by staining for CD31 (PECAM-1) and BS1 (lectin), as well as, laminin and col-IV. MS1 cells were highly positive for the endothelial cell markers, CD31 and BS1 (Figure 3A). RT-PCR analysis detected the expression of laminin-β1 and col-IV mRNAs in MS1 cells and purified primary murine islets, but not in βTC3 cells (Figure 3B). The expression of ECM proteins was further validated by immunofluorescence (IF) staining showing strong col-IV and laminin expression in MS1 cells (Figure 3C). The ability of MS1 cells to produce col-IV and laminin and the absence of ECM proteins in βTC3 cells cultured alone prompted us to examine whether laminin and col-IV are found in MS1-induced PIs. Z-stack confocal imaging showed clear staining of laminin and col-IV in and around the surface of the PIs (Figure 3D). Nonconsecutive z-stack images showed diffused col-IV staining, while laminin staining exhibited a more punctuated pattern (Figure 3E, Video S1). No staining was observed in PIs incubated with secondary antibodies only (data not shown).

**Figure 4:**
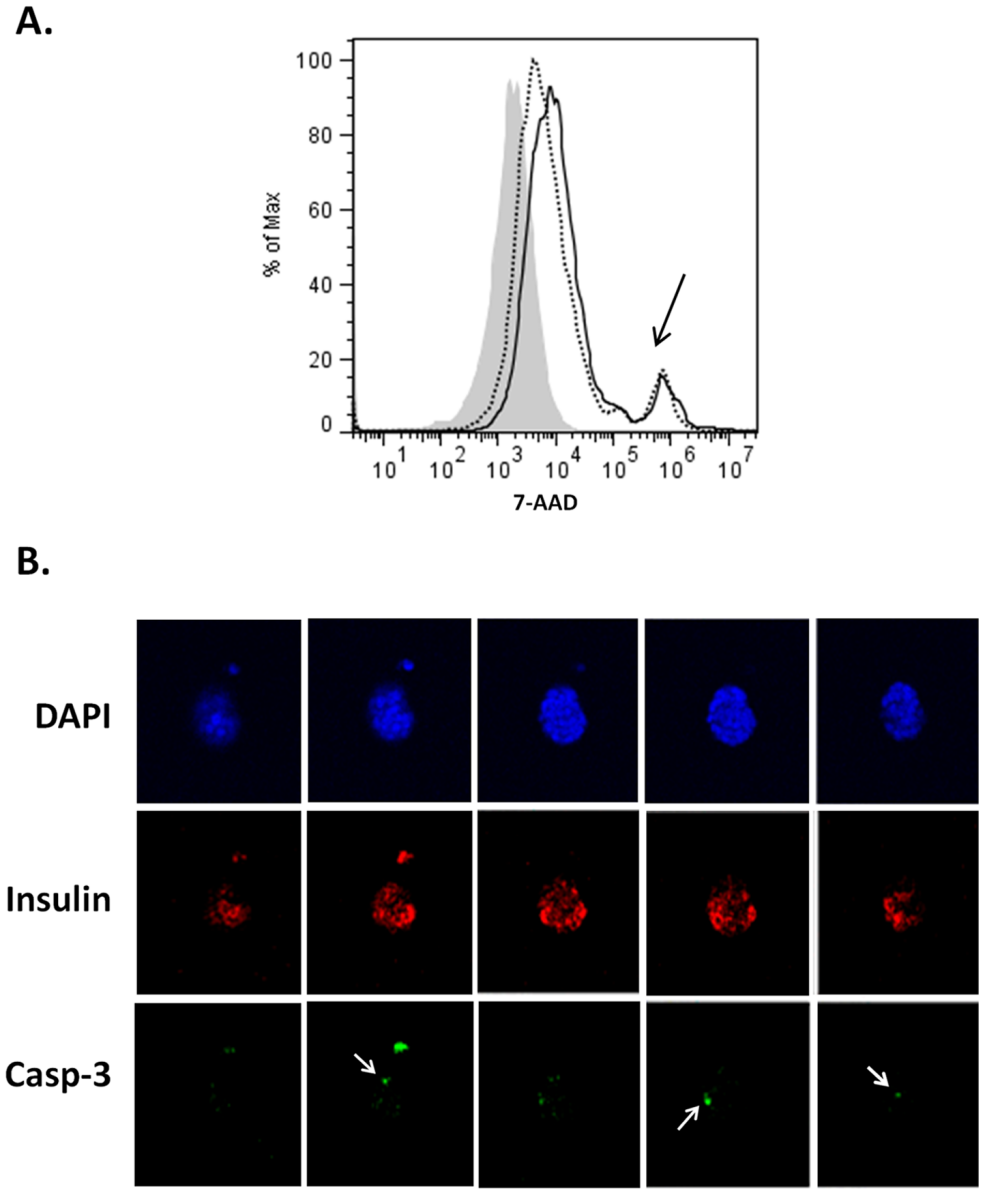
Evaluation of β cell loss in MS1-induced PIs. ***A.*** FACS staining for 7-AAD viability dye of monolayers (dotted line) and PIs (solid line). Grey histogram unstained control. Black arrow show 7-AAD positive cells. ***B.*** Non-consecutive z-stack confocal images of a 14 d old PI. Blue- DAPI, Red- Insulin, Green- cleaved caspase-3. White arrows point to caspase-3 positive cells.

PI formation may lead to increased β cell death over time [23]. Such an increase is attributed to reduced proliferation and increased apoptosis of insulinoma cells in PIs in comparison with monolayers. Therefore, we examined whether β cell death was increased in PIs when compared with monolayer cells. FACS analysis using the viability dye 7-AAD showed low and similar levels of β cell death in PIs and βTC3 monolayers (Figure 4A). Confocal imaging of intact 14 d old PIs did not reveal the presence of a necrotic core while showing strong and ubiquitous insulin staining throughout the PIs (Figure 4B). Similarly, staining for cleaved caspase-3, a marker of cell apoptosis, showed the presence of scattered apoptotic cells in the PIs which were not confined to the PI core, further demonstrating the absence of cell death at the PI core (Figure 4B, Video S2). Taken together, these data suggest MS1-induced PIs contain newly deposited ECM proteins and do not display an increase in β cell death compared to monolayer cultures, while exhibiting an intact insulin-positive PI core.

## Discussion

PIs are an important tool for the study of β cell physiology in a 3-D conformation [24]. In contrast to monolayer β-cells, PIs represent a more native structure for β cell cultures capable of improving β cell function. This improvement is partially attributed to enhanced cell-cell contact leading to increased glucose sensing, insulin production, and insulin release [4], [5], [6], [7], [8], [9]. Despite their usefulness, PI formation requires specialized culture conditions and extensive cell manipulations. Here we report a method for rapid and spontaneous induction of free-floating PIs by co-culturing MS1 and βTC3 cells. MS1-induced PIs show increased insulin synthesis and improved glucose responsiveness, do not differ significantly in the percentage of apoptotic cells compared to monolayer cells 14d post co-culture, and are positive for ECM proteins normally found in native islets. This novel method of PI formation provides a new tool for the study of β cell physiology in a 3-D conformation, while improving glucose sensing. PIs offer a convenient and rapid tool for examining the direct interactions between iECs and β-cells in vitro.

Current methods of PI formation require extensive cellular and mechanical manipulations of β-cells, thus limiting their use [4], [8], [13]. In our hands, co-culturing of MS1 cells together with βTC3 cells resulted in a rapid and spontaneous formation of free-floating PIs, as early as, 72 h in culture. This relatively short duration represents an improvement over previous methods requiring 7–14 d or longer for PI formation [4], [8], [13]. The spontaneous detachment of iEC-induced PIs provides an additional advancement over previous methods, by eliminating the need for mechanical detachment. PI detachment may be partially mediated by differences in the rate of cell division between iECs and β-cells. MS1 cells show a high turnover rate allowing the cells to reach a near 100% confluency by 24–48 h in culture. In contrast, βTC3 cells require 72–96 h before reaching full confluency. This difference in cell proliferation may limit the available area for βTC3 expansion, thus promoting the formation of β cell clusters and subsequent detachment of free-floating PIs. Indeed, the addition of βTC3 cells to a fully confluent MS1 monolayer results in the formation of PIs similar to those formed with mixed cultures, albeit at a faster rate (48–72 h, data not shown).

Both iECs and the ECM are an integral part of the islet [25]. The islet endothelium is a source of ECM in the islet [26]. In vitro culturing of β-cells on ECM-coated surfaces can increase β cell function and improve insulin production and glucose responsiveness [18]. Here we show that MS1, but not βTC3 cells, can produce laminin and col-IV in vitro. Both proteins were found in and around the PIs suggesting the continuous deposition of ECM proteins during PI formation. The punctuated pattern of laminin deposition suggests that the process of ECM deposition differs from that of a native islet and is most likely mediated by direct cell-cell contact between βTC3 and MS1 cells during PI formation. The accumulation of ECM proteins may further improve β cell contact and support cell adhesion, both of which can improve β cell function over time [27]. Interestingly, PIs did not include MS1 cells. The absence of MS1 cells in the PIs may relate to the relatively fast detachment of the PIs from the surface of the culture plate. Alternatively, the ability of MS1 cells to produce VEGF in an autocrine manner (data not shown), may preserve MS1-MS1 contact, thereby preventing the attachment of MS1 cells to the PI surface. In summary, PIs induced by iECs show the deposition of native ECM proteins in and around the PIs. This phenomenon offers a new method for the inclusion of key ECM proteins involved in β cell function and differentiation.

PI formation improves β cell function overtime [4], [5], [6], [7], [8], [9]. In our hands, MS1-induced PIs included an increased proportion of insulin-positive cells when compared with βTC3 monolayers. This increase was associated with enhanced insulin gene transcription and improved insulin secretion in response to escalating glucose concentrations. High passage insulinoma cells show reduced responsiveness to nutrient stimulation [28], which can be corrected following PI formation [4]. Hague-Evans et al. showed that high passage MIN6 cells with reduced insulin production and impaired glucose responsiveness exhibit improved cell function when cultured as PIs [4], while Kitsou-Mylona et al. showed that improved glucose sensing in PIs correlated with improved extracellular calcium sensing via the extracellular calcium-sensing receptors Car2 and Car4 [29]. Disruption of the Car pathway attenuated insulin secretion in response to glucose. Cell adhesion receptors such as E-cadherin and connexin are also involved in calcium oscillation, as the disruption of gap junctions can negatively affect insulin secretion of PIs [11], [30]. This enhanced cell-cell contact and increased calcium signaling may provide a mechanism whereby insulin expression and release is increased in PIs.

PI formation is often correlated with increased β cell death and reduced β cell proliferation. Luther et al. showed an overall increase in β cell death which was manifested by reduced proliferation and increased apoptosis of MIN6 cells in PIs when compared to monolayers [23]. Reers et al showed that MIN6 PIs exhibit an overall reduction in β cell proliferation [31]. These changes in β cell viability and proliferation represent a limitation for the use of PIs in the study of β cell physiology. In our model, β cell death was unaffected in PIs as we did not detect the formation of a necrotic core over time. MS1-induced PIs did not show an increased percentage of apoptotic cells compared to monolayer cultures 14 d after co-culture. These differences may be explained by the presence of a pro-β cell factor(s) produced by MS1 cells [21]. The identification of novel factors produced by iECs and the ability of these cells to induce the formation of PIs may offer a new way for induction of islet-like structures and improvement of β cell function in models involving primary β cell formation, such as stem cell-derived β cells.

In this report we describe a novel method for the rapid and spontaneous induction of PIs using co-cultures of MS1 and βTC3 insulinoma cells. iEC-derived PIs have integrated ECM proteins and show improved insulin production, enhanced glucose responsiveness and improved GSIS; offering an innovative tool for the study of β cell-iEC interactions in vitro.

## Supporting Information

Video S1 **3-D reconstruction of consecutive z-stack images of βTC3 PIs showing deposition of laminin and collagen IV in and around the islet as described in** **Figure 3E** **.** Blue- DAPI, Red- Insulin, Green- col-IV, White- laminin
(WMV)

Video S2 **3-D reconstruction of consecutive z-stack images of βTC3 PIs for examining β cell loss as described in** **Figure 4B** **.** Blue- DAPI, Red- Insulin, Green- cleaved caspase-3.
(WMV)

## References

[1] Paraskevas S, Maysinger D, Wang R, Duguid TP, Rosenberg L (2000) Cell loss in isolated human islets occurs by apoptosis. Pancreas 20: 270–276. 10766453 10.1097/00006676-200004000-00008

[2] Nanji SA, Shapiro AM (2006) Advances in pancreatic islet transplantation in humans. Diabetes Obes Metab 8: 15–25. 16367878 10.1111/j.1463-1326.2005.00476.x

[3] Rosenberg L, Wang R, Paraskevas S, Maysinger D (1999) Structural and functional changes resulting from islet isolation lead to islet cell death. Surgery 126: 393–398. 10455912

[4] Hauge-Evans AC, Squires PE, Persaud SJ, Jones PM (1999) Pancreatic beta-cell-to-beta-cell interactions are required for integrated responses to nutrient stimuli: enhanced Ca2+ and insulin secretory responses of MIN6 pseudoislets. Diabetes 48: 1402–1408. 10389845 10.2337/diabetes.48.7.1402

[5] Luther MJ, Hauge-Evans A, Souza KL, Jorns A, Lenzen S, et al (2006) MIN6 beta-cell-beta-cell interactions influence insulin secretory responses to nutrients and non-nutrients. Biochem Biophys Res Commun 343: 99–104. 16529716 10.1016/j.bbrc.2006.02.003

[6] Persaud SJ, Arden C, Bergsten P, Bone AJ, Brown J, et al (2010) Pseudoislets as primary islet replacements for research: report on a symposium at King's College London, London UK. Islets 2: 236–239. 21137597 10.4161/isl.2.4.12557

[7] Roderigo-Milne H, Hauge-Evans AC, Persaud SJ, Jones PM (2002) Differential expression of insulin genes 1 and 2 in MIN6 cells and pseudoislets. Biochem Biophys Res Commun 296: 589–595. 12176022 10.1016/s0006-291x(02)00913-0

[8] Lock LT, Laychock SG, Tzanakakis ES (2011) Pseudoislets in stirred-suspension culture exhibit enhanced cell survival, propagation and insulin secretion. J Biotechnol 151: 278–286. 21185337 10.1016/j.jbiotec.2010.12.015

[9] Kelly C, Guo H, McCluskey JT, Flatt PR, McClenaghan NH (2010) Comparison of insulin release from MIN6 pseudoislets and pancreatic islets of Langerhans reveals importance of homotypic cell interactions. Pancreas 39: 1016–1023. 20467348 10.1097/MPA.0b013e3181dafaa2

[10] Guo-Parke H, McCluskey JT, Kelly C, Hamid M, McClenaghan NH, et al (2012) Configuration of electrofusion-derived human insulin-secreting cell line as pseudoislets enhances functionality and therapeutic utility. J Endocrinol 214: 257–265. 22685334 10.1530/JOE-12-0188

[11] Squires PE, Hauge-Evans AC, Persaud SJ, Jones PM (2000) Synchronization of Ca(2+)-signals within insulin-secreting pseudoislets: effects of gap-junctional uncouplers. Cell Calcium 27: 287–296. 10859595 10.1054/ceca.2000.0117

[12] Maillard E, Sencier MC, Langlois A, Bietiger W, Krafft M, et al (2009) Extracellular matrix proteins involved in pseudoislets formation. Islets 1: 232–241. 21099277 10.4161/isl.1.3.9754

[13] Cavallari G, Zuellig RA, Lehmann R, Weber M, Moritz W (2007) Rat pancreatic islet size standardization by the “hanging drop” technique. Transplant Proc 39: 2018–2020. 17692680 10.1016/j.transproceed.2007.05.016

[14] Olsson R, Carlsson PO (2006) The pancreatic islet endothelial cell: emerging roles in islet function and disease. Int J Biochem Cell Biol 38: 492–497. 16162421 10.1016/j.biocel.2005.06.021

[15] Johansson M, Mattsson G, Andersson A, Jansson L, Carlsson PO (2006) Islet endothelial cells and pancreatic beta-cell proliferation: studies in vitro and during pregnancy in adult rats. Endocrinology 147: 2315–2324. 16439446 10.1210/en.2005-0997

[16] Jiang FX, Naselli G, Harrison LC (2002) Distinct distribution of laminin and its integrin receptors in the pancreas. J Histochem Cytochem 50: 1625–1632. 12486084 10.1177/002215540205001206

[17] Stendahl JC, Kaufman DB, Stupp SI (2009) Extracellular matrix in pancreatic islets: relevance to scaffold design and transplantation. Cell Transplant 18: 1–12. 19476204 10.3727/096368909788237195 PMC2724969

[18] Daoud J, Petropavlovskaia M, Rosenberg L, Tabrizian M (2010) The effect of extracellular matrix components on the preservation of human islet function in vitro. Biomaterials 31: 1676–1682. 20015544 10.1016/j.biomaterials.2009.11.057

[19] Arbiser JL, Moses MA, Fernandez CA, Ghiso N, Cao Y, et al (1997) Oncogenic H-ras stimulates tumor angiogenesis by two distinct pathways. Proc Natl Acad Sci U S A 94: 861–866. 9023347 10.1073/pnas.94.3.861 PMC19604

[20] Tal M, Thorens B, Surana M, Fleischer N, Lodish HF, et al (1992) Glucose transporter isotypes switch in T-antigen-transformed pancreatic beta cells growing in culture and in mice. Mol Cell Biol 12: 422–432. 1729614 10.1128/mcb.12.1.422-432.1992 PMC364137

[21] Olsson R, Carlsson PO (2006) The pancreatic islet endothelial cell: emerging roles in islet function and disease. Int J Biochem Cell Biol 38: 710–714. 16607697 10.1016/j.biocel.2006.02.004

[22] Akirav EM, Baquero MT, Opare-Addo LW, Akirav M, Galvan E, et al (2011) Glucose and inflammation control islet vascular density and beta-cell function in NOD mice: control of islet vasculature and vascular endothelial growth factor by glucose. Diabetes 60: 876–883. 21307078 10.2337/db10-0793 PMC3046848

[23] Luther MJ, Davies E, Muller D, Harrison M, Bone AJ, et al (2005) Cell-to-cell contact influences proliferative marker expression and apoptosis in MIN6 cells grown in islet-like structures. Am J Physiol Endocrinol Metab 288: E502–509. 15479950 10.1152/ajpendo.00424.2004

[24] Ogata T, Park KY, Seno M, Kojima I (2004) Reversal of streptozotocin-induced hyperglycemia by transplantation of pseudoislets consisting of beta cells derived from ductal cells. Endocr J 51: 381–386. 15256786 10.1507/endocrj.51.381

[25] Paraskevas S, Duguid WP, Maysinger D, Feldman L, Agapitos D, et al (1997) Apoptosis occurs in freshly isolated human islets under standard culture conditions. Transplant Proc 29: 750–752. 9123509 10.1016/s0041-1345(96)00452-6

[26] Nikolova G, Lammert E (2003) Interdependent development of blood vessels and organs. Cell Tissue Res 314: 33–42. 12898210 10.1007/s00441-003-0739-8

[27] Daoud J, Rosenberg L, Tabrizian M (2010) Pancreatic islet culture and preservation strategies: advances, challenges, and future outlook. Cell Transplant 19: 1523–1535. 20719076 10.3727/096368910X515872

[28] Josefsen K, Sorensen LR, Buschard K, Birkenbach M (1999) Glucose induces early growth response gene (Egr-1) expression in pancreatic beta cells. Diabetologia 42: 195–203. 10064100 10.1007/s001250051139

[29] Kitsou-Mylona I, Burns CJ, Squires PE, Persaud SJ, Jones PM (2008) A role for the extracellular calcium-sensing receptor in cell-cell communication in pancreatic islets of langerhans. Cell Physiol Biochem 22: 557–566. 19088438 10.1159/000185540

[30] Rogers GJ, Hodgkin MN, Squires PE (2007) E-cadherin and cell adhesion: a role in architecture and function in the pancreatic islet. Cell Physiol Biochem 20: 987–994. 17982281 10.1159/000110459

[31] Reers C, Hauge-Evans AC, Morgan NG, Wilcox A, Persaud SJ, et al (2011) Down-regulation of proliferation does not affect the secretory function of transformed beta-cell lines regardless of their anatomical configuration. Islets 3: 80–88. 21505275 10.4161/isl.3.3.15428

